# Text-Book of Hygiene

**Published:** 1894-12

**Authors:** 


### Book Reviews.

---

Text-Book of Hygiene: A Comprehensive Treatise on the Principles and Practice of Preventive Medicine From an American Standpoint. By George H. Rohe, M. D., Professor of Therapeutics, Hygiene, and Mental Diseases in the College of Physicians and Surgeons, Baltimore; Superintendent of the Maryland Hospital for the Insane; Member of the American Public Health Association; Foreign Associate of the Societe Francaise d'Hygiene, etc. Third Edition, Thoroughly Revised and Largely Rewritten, with Many Illustrations and Valuable Tables. Royal Octavo, 553 Pages. Cloth, $3.00. Philadelphia: The F. A. Davis Co., Publishers, 1915 and 1916 Cherry street.

In this new edition of this comprehensive treatise, we naturally expected to find some mention of inoculation as a preventive for yellow fever, so successfully performed by Dr. Domingos Freire, in Brazil, and recently endorsed by the International Health Congress at Buda-Pesth, Hungary. It is rather too much to expect mention of the inoculation of anti-toxine for the prevention or cure of diphtheria, although it has been practiced for one or two years and was also endorsed at Buda-Pesth. We would like to see some book on hygiene give more space to sexual hygiene, and treat of it in some such manner as done by Dr. E. L. Keyes in his work on "Genito-Urinary Diseases with Syphilis."

Dr. Rohe traces the history of syphilis, and delineates in graphic lines the ravages of venereal diseases in armies, and closes by saying: "An inspection at regular intervals not only of public prostitutes, but also of the soldiers themselves, and segregation of the infected in hospitals until the infective period is past, will do more to limit the spread of venereal diseases than all other preventive measures, public or private put together."

This is a strong statement, and is so positive as to make the influence of early education and training at home and in school seem to be of little influence.

Rather let us trust to perfect continence in youth and early marriage with an untainted manhood, a vigorous constitution and a pure conscience. In the words of Prof. Theophilus Parvin:*

* "The Conduct of the Individual Medical Student."—*Medical and Surgical Reporter*, Nov. 17, 1894.

"Wise opinions and judicious counsels on your part may do much to lessen this great evil of the ages, while your own lives of continence will give additional strength to your efforts in accomplishing this end."

There can be no doubt that to perfect ignorance of sexual hygiene is to be traced much venereal disease.

And as to the regulation of prostitution, Parvin says: "The certificates from doctors, whether appointed by the government, or volunteers, who for paltry pay open wider the doors of illegitimate indulgence by taking away, or lessening the fear of infection, are often—usually, indeed, not worth the paper upon which they are written, as securing probable immunity from disease. In Berlin, there are eight thousand licensed prostitutes and thirty thousand clandestine ones. I recently asked a medical acquaintance in that city whose practice is largely in venereal diseases, by which class his male patients were more frequently infected, and his reply was, by the former.

"Prostitution prevents the normal growth of population, for it sterilizes thousands and tens of thousand of women who are its victims; it delays or prevents marriages and when men who have been engaged in debauchery turn from their dissolute life and marry, they may carry disease to the nuptial couch, so that their wives are sterile or give birth to syphilitic children."

The army and navy of the United States should be composed of good husbands and men of the greatest temperance and self-control.

The articles on quarantine, and marine hygiene, have been rewritten or revised by experts and bring these important subjects up to date.

The work of the sanitarian is beginning to be fully appreciated and this book, dealing in points of general interest, should be generally read and studied. An important help to the student is to be found in the questions appended to the chapter.

We note with special pleasure the remark as to hydrophobia: "Inoculation with the attenuated virus protects the bitten individual against the fatal outbreak of the general disease." But the important statistics recently published in the reports of the Pasteur institutes might have been given.

J. McF. G., Jr.

THE MEDICAL NEWS VISITING LIST for 1895: Weekly (dated, for 30 patients); Monthly (undated, for 120 patients per month); Perpetual (undated, for 30 patients weekly per year); and Perpetual (undated, for 60 patients weekly per year). The first three styles contain 32 pages of data and 160 pages of blanks. The 60-Patient Perpetual consists of 256 pages of blanks. Each style in one wallet-shaped book, with pocket, pencil and rubber. Seal Grain Leather, $1.25. Philadelphia: Lea Brothers & Co., 1894.

*The Medical News Visiting List* for 1895 has been thoroughly revised and brought up to date in every respect. The text portion (32 pages) contains the most useful data for the physician and surgeon, including an alphabetical Table of Diseases, with the most approved Remedies, and a Table of Doses. It also contains sections on Examination of Urine, Artificial Respiration, Incompatibles, Poisons and Antidotes, Diagnostic Table of Eruptive Fevers, and the Ligation of Arteries. The classified blanks (160 pages) are arranged to hold records of all kinds of professional work, with memoranda and accounts. The selection of material in the text portion and the arrangement of the record blanks are the result of ten years of experience and special study. Equal care has been bestowed upon the mechanical execution of the book, and in quality of paper and in strength and beauty of binding nothing seems to be left wanting. When desired, a Ready Reference Thumb-letter Index is furnished, which is peculiar to this visiting list, and which will save manyfold its small cost (25 cents) in the economy of time effected during a year. In its several styles, *The Medical News Visiting List* adapts itself to any system of keeping professional accounts. In short, every need of the physician seems to have been anticipated in this invaluable pocket companion.

